# Improving Concussion Management by Including Driving Recommendations for Adolescents with Concussions: A Quality Improvement Project

**DOI:** 10.1097/pq9.0000000000000307

**Published:** 2020-05-28

**Authors:** Jonathan A. Santana, Rebecca Martinie, Jorge Gomez

**Affiliations:** From the * Department of Pediatrics, Section of Adolescent and Sports Medicine, Baylor College of Medicine, Houston, TX, USA; † Texas Children’s Urgent Care, Houston, TX, USA

## Abstract

**Introduction:**

Motor vehicle collisions are the leading cause of death in adolescents. A concussion is a common injury sustained by adolescents and may impair their driving abilities during the acute phase of recovery. Healthcare providers do not consistently perform counseling of adolescents regarding driving after a concussion. This quality improvement project’s goal was to increase the incidence of primary care sports medicine physicians providing driving recommendations to 75% of adolescents who suffered from concussions.

**Methods:**

Between August 2017 to August 2018, a “smart phrase” implemented in the electronic health record system reminded providers during office visits to provide driving recommendations to patients 15 years old and older who presented to the sports medicine clinic for evaluation of concussion. Performing monthly retrospective chart reviews determined the frequency of patients who received driving recommendations during the initial visit.

**Results:**

We achieved the goal of providing driving recommendations to 75% of concussed patients by the second month. This progress was maintained through the remainder of the year, except for 1 month (December). Forty-three percent of patients with concussions evaluated met inclusion criteria, and of those, 48% were actively driving before their concussion. The most common medical reason for restricting driving was vestibular or ocular dysfunction.

**Conclusion:**

This quality improvement project showed that providing driving instructions to concussion patients by implementing a smart phrase into the electronic health record system was impactful and sustainable.

## INTRODUCTION

Sports-related concussions (SRCs) are a form of traumatically induced transient disturbances of brain function that involve a complex physiological process.^1^ Annually, 1–1.8 million SRCs occur in youth younger than 18 years old in the United States, and a subset of approximately 400,000 SRCs arise in high school athletes.^2^ One of the significant effects that these injuries impose on adolescents is to render daily tasks, including driving a motor vehicle, more challenging to perform.

Motor vehicle collisions are the leading cause of death in adolescents, accounting for 1,830 motor vehicle deaths in individuals 15–20 years of age in 2017.^3,4^ Motor vehicle collisions are also the leading cause of death in collegiate athletes.^5^ Driving is an inherently dangerous activity, especially for young, inexperienced drivers.^6,7^ A challenging and complex task, driving requires high cognitive skills as well as visual coordination and motor skills.^8^ These skills are affected by an acute concussion.^9,10^ Recent consensus guidelines for acute management of sports concussion include recommending symptom-limited cognitive and physical rest during the first 24–48 hours, followed by a gradual return to a regular daily routine, including the return to school, sports, and driving.^1,11^

Patients and their families seldom are counseled regarding the dangers of driving while symptomatic after sustaining a concussion. One study surveyed 81 patients regarding their recovery expectations after sustaining a mild traumatic brain injury, and almost 50% reported that they did not intend to change their driving habits after injury.^12^

Hence, counseling on this topic by providers remains inadequate. Lucas et al^13^ surveyed more than 300 physicians who manage SRCs and found that fewer than half indicated that they “almost always” counsel patients on driving after concussions. The most recent American Medical Society for Sports Medicine position statement for managing concussion in sports was the first position statement to address the importance of discussing return-to-driving with young athletes.^1^

As pediatric sports medicine specialists, it is incumbent to begin counseling patients regarding driving recommendations when concussed. Stuart et al^14^ showed that an electronic health record (EHR) could be used to communicate driving recommendations to adolescents with concussions. The purpose of our quality improvement (QI) project was to implement a “smart phrase” within the EHR to improve physicians’ communication of driving instructions to patients of driving age who presented with concussions. If appropriately implemented, this intervention will address a serious gap in our current education and management of pediatric patients who present with concussions.

## OBJECTIVE

The goal of our study was to increase healthcare providers’ implementation of driving instructions to patients 15 years of age and older who present at one of our clinics with a new concussion, and with the aim that by the end of 3 months, 75% of these patients would receive the recommendations during their initial visit.

## METHODS

The project leaders elected to use Plan-Do-Study-Act (PDSA), a standard methodology in healthcare QI projects.^15^ The 4-step cyclical methodology involves (1) planning an initial intervention to an identified problem; (2) implementing the intervention; (3) studying the outcomes; and (4) acting to sustain or improve the intervention.^15,16^ There were 3 PDSA cycles throughout the project.

Primary care sports medicine physicians at Texas Children’s Hospital, who are in the Section of Adolescent Medicine and Sports Medicine, Department of Pediatrics, Baylor College of Medicine, performed this QI project. The Section has 6 sports medicine physicians evaluating concussions in 6 locations throughout Houston and the surrounding areas. Concussions account for roughly 20% of all patient visits seen in sports medicine over the past 2 years (2,300 visits/y). The hospital uses Epic Systems (Verona, Wis.) as its EHR. All providers in the Section participated in the project. Texas Children’s Maintenance of Certification steering committee approved the project for Maintenance of Certification credit. As adolescents may obtain a driving permit at age 15 in Texas, we included patients who were 15 years of age and older presenting for their initial evaluation for a concussion.

## STUDY DESIGN

Before implementation of the QI project, the project leaders held several education sessions to develop the smart phrase, as well as to allow providers the opportunity to give input and learn how to incorporate the smart phrase into the patient’s instruction section of the EHR.

All patients diagnosed with a concussion receive general instructions, including education regarding the diagnosis, school accommodations, and activity recommendations. In addition to the traditional instructions, the project leaders developed an EHR “concussion-driving” smart phrase which contained a hard stop where the provider would be required to give driving instructions to those patients 15 years of age and older, before the closure of the patient instructions. The recommendations for return-to-drive included the following options for the patient: cleared to drive, should not drive until after next visit, should not drive if symptoms worsen, should drive only short distances, should not drive at night, and not applicable as the patient does not drive. Providers also had a free-text option if needed. The patient would then receive these instructions upon checking out or through their online portal (eg, MyChart, Epic Systems, Verona, Wis.).

## BASELINE DATA COLLECTION

Initial data collection occurred in August 2017 to determine a baseline of how many patients received driving recommendations.

### PDSA 1

Implementation of the “concussion driving” smart phrase began on September 1, 2017, and data collection occurred over 12 months, September 2017–August 2018. After the first month of implementing the smart phrase, the physicians’ input was requested regarding ease of using the smart phrase and any other issues related to its use.

### PDSA 2

In the second PDSA cycle, providers documented their clinical reasons for placing driving restrictions in the patient’s record.

### PDSA 3

During the third PDSA cycle, several of the patients with new concussions were considered ready to be cleared and given the “return-to-play” protocol, instead of the concussion-driving smart phrase. These patients’ concussions had resolved clinically. The project leaders considered these patients as not needing driving recommendations or restrictions, so they were cleared to begin their return-to-play protocol; these patients were removed from analysis starting in the fifth month.

## DATA COLLECTION

Every month, providers performed retrospective reviews of their charts and inputted the following information: the number of total new concussions for that month, if the patient met inclusion criteria, their driving status, the smart phrase usage, and, if appropriate, the reason for driving restriction. The providers alone were responsible for the accuracy of their data. Providers also documented if patients were cleared on their initial visit and did not receive the concussion-driving smart phrase on their after-visit summary. Project leaders reviewed the charts of the concussed patients and inputted any missing or incorrect information.

The percentage of eligible subjects who received driving recommendations was calculated after the completion of all monthly chart reviews. We analyzed the data and plotted it on a run chart. Presentation and discussion of the results occurred during monthly faculty meetings. To determine the sustainability of the intervention, we completed a chart review for all new concussed patients 6 months postcompletion (February 2019) of the project.

## RESULTS

During the 1-month baseline period, a total of 38 new patients with concussions presented to the sports medicine clinic, with 21 patients meeting inclusion criteria. Of those 21 patients, only 11% received any driving recommendations.

Throughout the department, 740 new concussion evaluations occurred, of which 321 patients met the criteria for inclusion in the study. On average, 27 patients met criteria per month, with a range of 4–53 patients. Overall, 43% of the concussions evaluated were in individuals 15 years and older (Table 1); of those, 48% were actively driving before sustaining their concussion (Table 2). Providers documented clinical reasoning for driving restrictions in 44% of the charts.

**Table 1.**
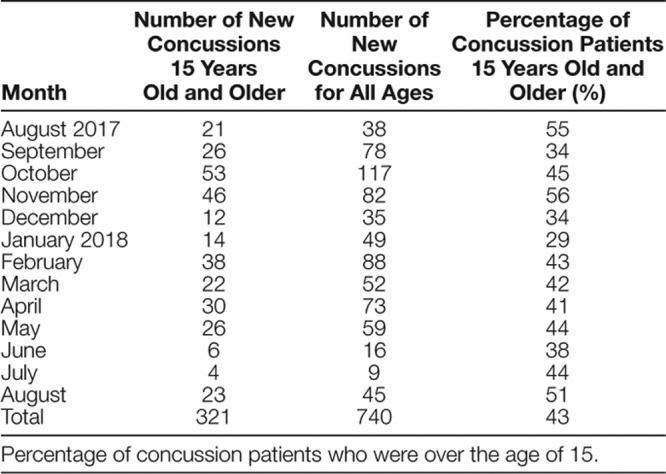
Shows the Number of New Concussions Seen Monthly and the Percentage of Patients Who Met Inclusion Criteria

**Table 2.**
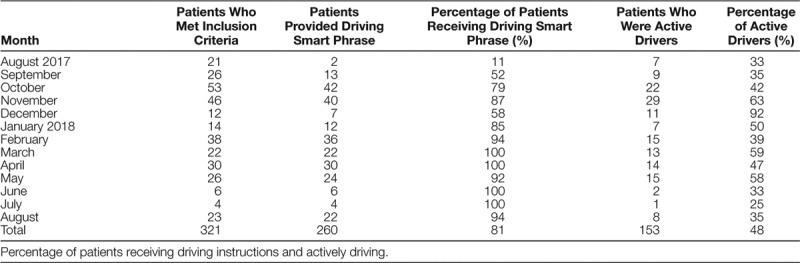
Percentage of Patients Who Were Provided Smart Phrase and Actively Driving During the Project

**Fig. 1.**
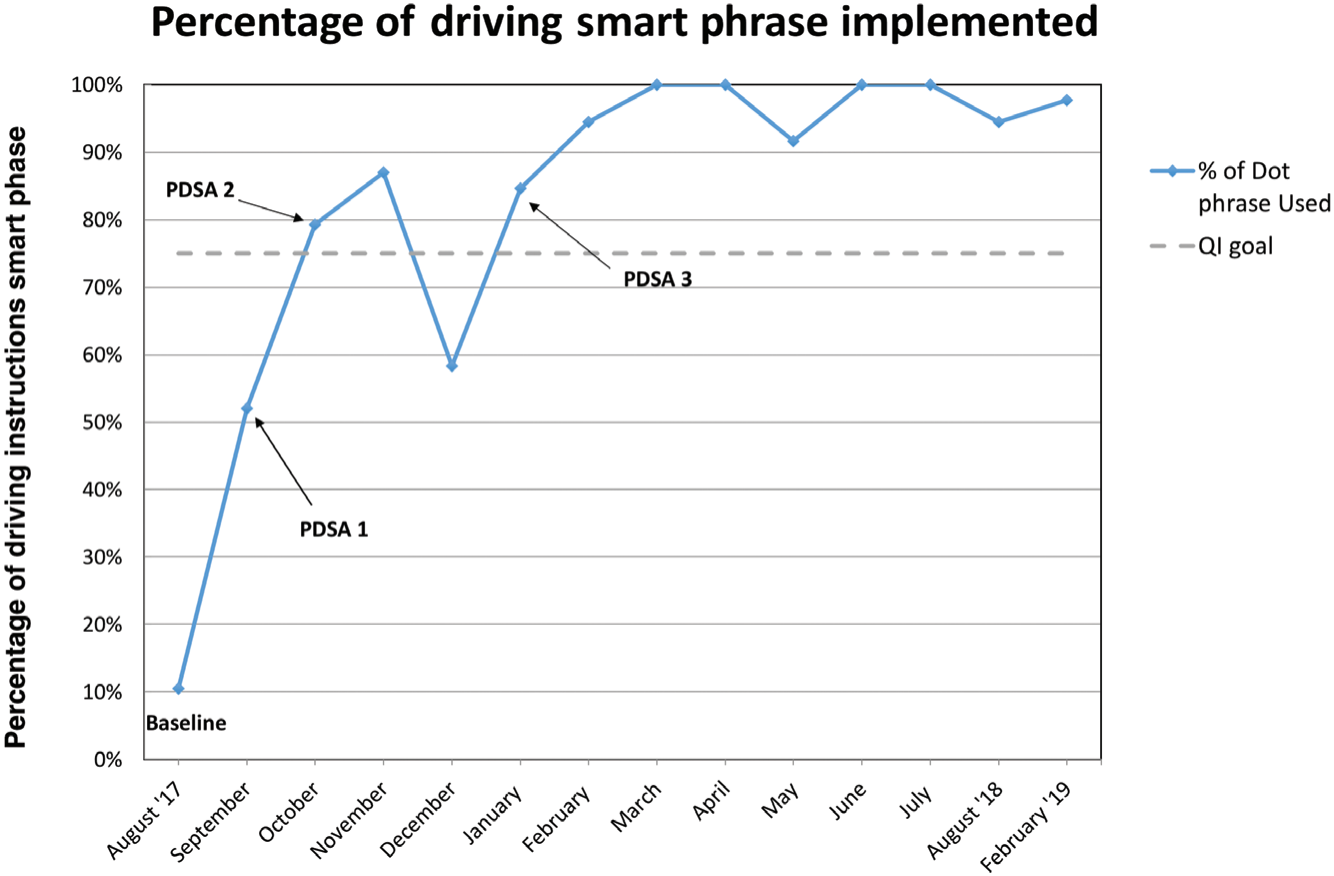
The percentage of eligible patients receiving the driving instructions smart phrase and the timing of the different PDSA cycles.

The goal of providing driving recommendations to 75% of new patients with concussions was achieved by the second month after the implementation of the smart phrase (Fig. 1, Table 2). We maintained this improvement during every subsequent month except month 4 (December), which recorded a decrease to 58%.

During the second PDSA cycle, providers added their reason for issuing the driving restriction. Of those patients who were restricted or had modifications from driving, vestibular dysfunction (46%) and saccadic eye movement deficiency (15%) were the top 2 reasons. Other reasons included convergence deficiency, dizziness, sensitivity to light, or blurred vision.

Six months after completion of the project (February 2019), a chart review for new concussion visits was performed for that month and revealed that 94 total new patients with concussions presented to sports medicine clinics, with 53 meeting inclusion criteria. Providers included the concussion-driving recommendations in 98% (52/53) of those patients’ instructions.

## DISCUSSION

Overall, the goal was reached within a few months and maintained 18 months after initiating the QI project. We attribute the success of the project to careful design, proactive providers, and general ease of use. A noticeable decline to 58% occurred in December due to a few patients being cleared on their initial visit, thus receiving the return-to-play protocol rather than the concussion smart phrase. This decline occurred before the third PDSA cycle, so we did not exclude the patients from analysis. When corrected for those patients, we would have met the goal for the month, as well.

Our study complements what Stuart et al^14^ previously showed when using a multidimensional active approach. EHR implementation had the most significant effect on changing providers’ ability to deliver driving recommendations to concussion patients.^14^ Monthly email reminders and discussions of the overall progress of the project during department meetings were sufficient to maintain the use of the smart phrase. Including patients who were 15 years of age allowed us to identify a subgroup of young drivers that have not been previously included in other QI projects.

Recommendations based on earlier studies indicate that concussed individuals should abstain from driving for the first 24–48 hours or until they feel safe enough to drive.^8–10,12,17,18^ Even with this increased awareness, understanding of the best objective way to determine return-to-drive criteria is poor. This study included providers’ clinical reasoning for recommending driving restrictions.

Emerging information indicates persistent driving impairment for the 24 hours after sustaining a concussion. Schmidt et al^17^ showed that even after the clinical resolution of a concussion, drivers were more likely to have lane excursions and drive onto the shoulder of the lane compared to controls. Preece et al^10^ showed impairment in hazard perception in concussed adults. There are also data indicating possible long-term effects in driving ability including more frequent aberrant driving behaviors, dangerous driving, and a higher risk of collisions.^19^ Clinicians need to weigh the evidence and make decisions on how to advise their patients on a case-by-case basis.^18^

Given that there are no evidence-based guidelines on driving restrictions, it was essential to document the providers’ clinical concern for their recommendations or restrictions. The most common reason for restricting patients was vestibular-ocular dysfunction, which is seen in almost 80% of pediatric patients with concussion.^20^ All providers in the clinic use validated vestibular-ocular-motor screening to evaluate for vestibular-ocular dysfunction.^21^ The vestibular-ocular-motor screening is a set of physical tests used to identify dysfunction at the systems responsible for vision, head movement, and balance integration. Dysfunction in these systems can affect driving, as these patients will have symptoms with rapid eye movements or quick head-turning. It is impractical to test an individual’s driving performance after an SRC directly. Several studies use other indicators such as driving simulators, which are available only in specialized centers. Computer neurocognitive tests have been used to determine reaction time and may influence a physician’s decision on return-to-driving recommendations.^22^ However, there are several issues with the reliability of computer neurocognitive tests.^23–25^ A vestibular-ocular screen can easily be performed in the clinic and may be the preferred clinical determinate of whether or not a patient is clear to return to drive.

As noted earlier, we excluded patients who cleared at their first visits from the analysis starting in January. We considered this decision to be appropriate because these patients are, by definition, asymptomatic and, thus, do not require recommendations on driving. It is not uncommon for patients to be cleared at their initial sports medicine visit, as most patients are referred and, hence, have already been evaluated by their primary care physician or team doctor.

The impact was significant as 43% of new patients with concussions were old enough to be included, and almost half of those patients were active drivers. Many families had not thought about how a concussion affects driving before our discussion with them. This fact emphasizes the importance of adding this conversation to a visit for concussion. Parents were appreciative of the counseling, and providers stated that it did not significantly lengthen the duration of the visit.

The study had certain limitations. Given that the study site is a teaching hospital, there are learners (medical students, residents, and fellows) in clinics who often are responsible for providing patients’ instructions for those patients they evaluate. Education regarding the implementation of the new smart phrase was provided to the sports medicine fellows. However, it was difficult to ensure that all learners were aware of using the smart phrase, as some of them are present for only 2- to 4-week rotations and are from various other institutions, thereby accounting for a decrease in usage. Another limitation was that the reason patients were restricted was not always clear from the providers’ documentation. Specific driving restrictions discussed with patients were not analyzed.

Furthermore, providers did not provide driving recommendations for individuals younger than 15, who may operate motorized bicycles, scooters, farming equipment, or all-terrain motor vehicles. Driving instructions for patients seen outside our Section, such as those presenting to the emergency department or other primary care providers, were not evaluated. Also, the study required providers to only document driving recommendations during the initial visit. We did not look into the duration for patients to be cleared to drive or the clinical reasoning for clearance.

After 1 year of data collection, this process has become well established in the sports medicine clinics. For the future, we are considering the expansion to other clinical sites that treat concussions, including the emergency department, urgent care facilities, and affiliated general pediatric clinics. It would also be worthwhile to evaluate the criteria providers use to determine readiness to return to driving.

## CONCLUSIONS

This QI project showed that a small intervention could have a significant impact on our patient population, given that almost half of our patients with concussions were of driving age. The intervention increased driving recommendations from 11% to 98% by the implementation of a smart phrase in the EHR system. This smart phrase provided valuable counseling and education to families and adolescent drivers after sustaining a concussion.

This intervention built on previous studies and showed it could be easily replicated in other practices that use EHR systems. It is also important to screen for vestibular-ocular dysfunction as screening could help determine initial driving recommendations and be used as clinical criteria for giving a patient clearance to drive. Future studies will need to address more uniform criteria to help clinically assess an individual’s ability to drive safely.

The topic of return-to-driving after sustaining a concussion continues to evolve, but it is paramount to encourage providers who manage patients with concussions to have this discussion. Concussions may place these young drivers at significant risk for further injury or death, or danger to other drivers.

## ACKNOWLEDGMENTS

The authors thank Dr. Lee Ligon of the Department of Pediatrics, Baylor College of Medicine, for editorial assistance.
